# Protein Kinase Cε Modulates Insulin Receptor Localization and Trafficking in Mouse Embryonic Fibroblasts

**DOI:** 10.1371/journal.pone.0058046

**Published:** 2013-03-01

**Authors:** David J. Pedersen, Barbara Diakanastasis, Jacqueline Stöckli, Carsten Schmitz-Peiffer

**Affiliations:** 1 Diabetes and Obesity Program, Garvan Institute of Medical Research, Sydney, New South Wales, Australia; 2 St Vincent’s Clinical School, Faculty of Medicine, University of New South Wales, Sydney, New South Wales, Australia; Tohoku University, Japan

## Abstract

We have previously shown that deletion of protein kinase C epsilon (PKCε) in mice results in protection against glucose intolerance caused by a high fat diet. This was in part due to reduced insulin uptake by hepatocytes and insulin clearance, which enhanced insulin availability. Here we employed mouse embryonic fibroblasts (MEFs) derived from wildtype (WT) and PKCε-deficient (PKCε^−/−^) mice to examine this mechanistically. PKCε^−/−^ MEFs exhibited reduced insulin uptake which was associated with decreased insulin receptor phosphorylation, while downstream signalling through IRS-1 and Akt was unaffected. Cellular fractionation demonstrated that PKCε deletion changed the localization of the insulin receptor, a greater proportion of which co-fractionated with flotillin-1, a marker of membrane microdomains. Insulin stimulation resulted in redistribution of the receptor in WT cells, while this was markedly reduced in PKCε^−/−^ cells. These alterations in insulin receptor trafficking were associated with reduced expression of CEACAM1, a receptor substrate previously shown to modulate insulin clearance. Virally-mediated reconstitution of PKCε in MEFs increased CEACAM1 expression and partly restored the sensitivity of the receptor to insulin-stimulated redistribution. These data indicate that PKCε can affect insulin uptake in MEFs through promotion of receptor-mediated endocytosis, and that this may be mediated by regulation of CEACAM1 expression.

## Introduction

Type 2 diabetes results in part from diminished insulin action in peripheral tissues such as skeletal muscle and liver, and in part from a failure of the pancreatic β-cells to compensate by increasing insulin secretion in response to elevated blood glucose levels [1]. In each case these defects are often mediated by increased lipid availability, and chronic activation of isoforms of the lipid-activated protein kinase C (PKC)^4^ family has been extensively associated with the development of insulin resistance in cellular and whole animal models and also in human studies [2]. Phosphorylation of insulin receptor substrate (IRS)-1 or IRS-2 on serine residues by PKCs may reduce subsequent tyrosine phosphorylation by the insulin receptor, leading to inhibition of proximal insulin signalling and hence to defects in glucose disposal [2]. In the liver, PKCε may also more directly inhibit insulin receptor activation itself [3].

We have previously shown that PKCε also plays a role in insulin availability, by affecting glucose-stimulated insulin release by β-cells and in addition by modulating insulin clearance [4]. Over 50% of newly-released insulin is cleared by the liver before entering the general circulation [5]. Although the reasons for this are not well-understood, mice deficient in PKCε (PKCε^−/−^ mice) exhibit reduced clearance of exogenous insulin, and the rate of insulin uptake by primary hepatocytes from these animals is reduced [4]. This was not associated with any defect in insulin signal transduction, suggesting that targeting the mechanism involved may represent a novel strategy for improving glucose homeostasis when insulin secretion and insulin action are compromised.

Insulin clearance by the liver has also been shown to be dependent on carcinoembryonic antigen-related cell adhesion molecule 1 (CEACAM1), a tumor suppressor belonging to the immunoglobulin superfamily. Mice expressing a dominant negative CEACAM1 mutant in liver developed hyperinsulinemia resulting from reduced insulin clearance due to impaired insulin receptor internalization [6]. CEACAM1 is a substrate of the receptor and may form part of a complex involved in the internalization of insulin bound to the insulin receptor [7].

We have examined this further in mouse embryonic fibroblasts (MEFs) generated from wild type (WT) and PKCε^−/−^ mice. We found that the absence of PKCε in these cells was associated with alterations in insulin receptor trafficking, and that this may be linked to the expression levels of CEACAM1.

## Materials and Methods

### Materials

General reagents of analytical grade were obtained from Sigma-Aldrich Pty. Ltd. (Sydney, NSW, Australia), Calbiochem (Sydney, NSW, Australia) and Invitrogen (Victoria, Australia) unless otherwise stated. The human insulin analogue Actrapid™ was from Novo Nordisk (Copenhagen, Denmark) and fluorescein isothiocyanate (FITC)-labelled insulin was from Molecular Probes (Eugene, Oregon). Complete Protease Inhibitor and PhosSTOP were from Roche Pty. Ltd. (Sydney, NSW, Australia). Details of antibodies used are given in Methods S1.

### Isolation of MEFs

Ethical approval for mouse studies was granted by the Garvan/St Vincent’s Hospital Animal Ethics Committee (Approval numbers 07/16, 10/23). PKCε^−/−^ mice were generated and genotyped as described previously [4]. PKCε^+/−^ mice on a pure C57Bl/6 background were mated and WT and PKCε^−/−^ MEFs were isolated from 10-day-old embryos by digestion with trypsin, after removal of visible organs. Cells were maintained in Dulbecco’s Modified Eagle Medium with 5 mM glucose, containing 10% (v/v) fetal calf serum and 1% (v/v) antibiotic/antimycotic solution (10,000 units/mL penicillin G, 10 mg/mL streptomycin, 25 pg/mL amphotericin B). Three independently generated pools of WT and PKCε^−/−^ MEFs were selected after genotyping.

### Determination of Insulin Uptake

Insulin uptake by MEFs was determined either by fluorescence microscopy or fluorescence-activated cell sorting (FACS). In each case, cells were serum-starved for 2 hours prior to insulin stimulation for 10 min using 100 nM FITC-labelled insulin, and surface bound insulin removed by acid washes. Full details are given in Methods S1.

### Membrane Structure and Fluidity

Gross membrane structure of MEFs was examined by transmission electron microscopy (TEM) after cells were fixed and incubated with osmium tetroxide [8]. To measure membrane fluidity, cells were stained with Laurdan and examined using 2-photon microscopy. Full details are given in Methods S1.

### Subcellular Fractionation

After serum starvation for 2 h, cells grown in 15 cm dishes were treated with or without 100 nM insulin for the indicated time. Media was aspirated and cells placed on ice and washed three times with ice-cold PBS. Subsequent procedures were carried out at 4°C. Cells were harvested in 1 ml fractionation buffer (250 mM Sucrose, 1 mM EDTA, 20 mM Hepes, pH 7.4, 10 mM NaF, 2 mM phenylmethylsulfonyl fluoride, 100 µM leupeptin, 2 mM benzamidine, 2 mM sodium orthovanadate and Complete Protease Inhibitor). Cells were disrupted by nitrogen cavitation (450 psi N_2_(g) for 15 min in a 50 mL chamber (Parr Instrument, Moline, IL). Lysates were collected and unbroken cells removed by centrifugation at 500 *g* for 10 min. The protein content of the supernatants was measured by Bradford assay (Biorad), and equal amounts of protein (500 µg) were loaded onto 15% (v/v) iodixanol (Opti-Prep, Axis-Shield PoC, Oslo, Norway) and centrifuged at 100,000 *g* for 4 h to form a continuous gradient. Fractions were removed starting from the top of the gradient for analysis by immunoblotting.

### Immunoblot Analysis

Following treatments as indicated, cells were washed three times with ice-cold PBS, harvested in RIPA lysis buffer, sonicated and centrifuged as previously [9]. Total protein in supernatants was determined by BCA assay (BioRad). Samples of equal protein content were subjected to SDS-PAGE using 7% or 10% NU-PAGE gels (Invitrogen) followed by immunoblotting, chemiluminescent detection and quantification using Image J [9].

### Adenovirus-mediated PKCε Re-expression

Recombinant adenoviruses for the expression of green fluorescent protein (GFP) alone or together with PKCε were generated and characterised using the pAdEasy system as described previously [10]. MEFs were infected with an amount of virus required to give 80–100% infection efficiency, determined by visualizing GFP expression using fluorescence microscopy 48 h post infection.

### Generation of Stable MEF Lines Re-expressing PKCε

Rat PKCε cDNA, a gift from G. Baier, University of Innsbruck, Austria [11], was subcloned into the retroviral vector pQXCIP (Invitrogen). This construct, or empty pQXCIP vector, was used to transfect PlatE cells. Concentrated virus stocks were prepared from media harvested 48 h post transfection and used to infect MEFs. Puromycin was used to select for cells stably expressing the retrovirus, while cells selected after infection with empty vector served as control.

## Results

### PKCε^−/−^ MEFs Display Reduced Insulin Internalization

**Figure 1:**
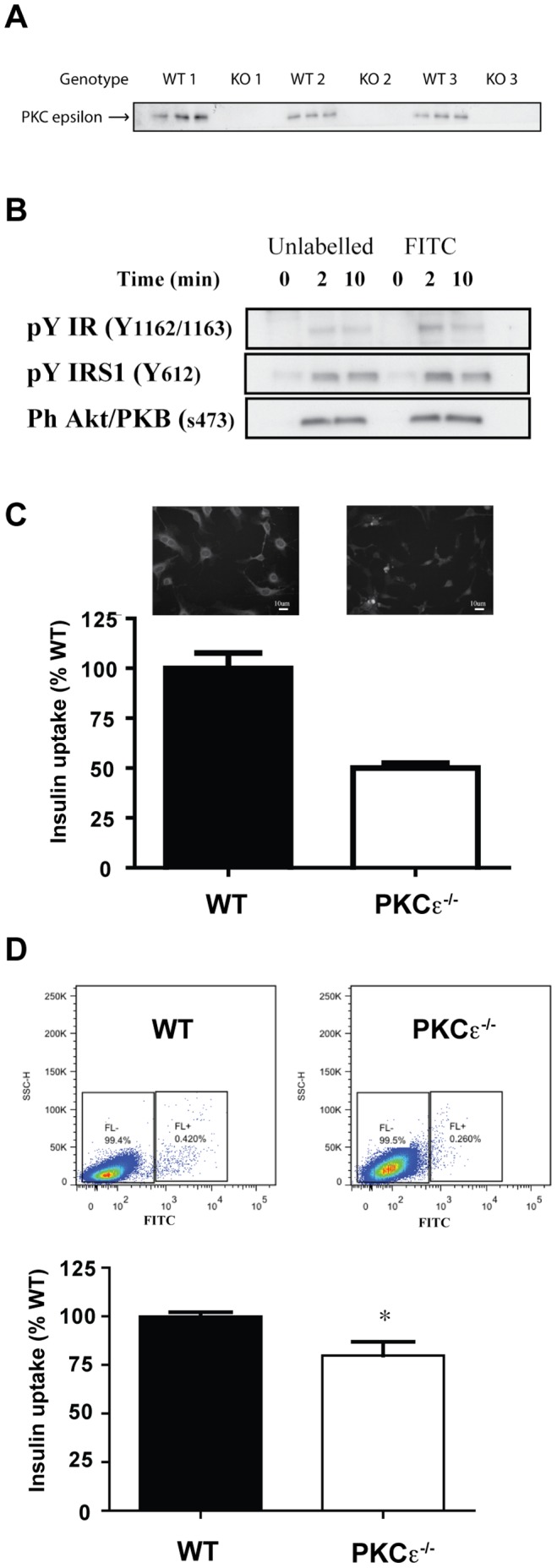
PKCε expression and receptor ligand uptake in WT and PKCε^−/−^ MEFs. A. PKCε expression in three pairs of MEFs was assessed in triplicate by immunoblotting, after cells were extracted and equal amounts of protein were subjected to SDS-PAGE. B. Activation of insulin signalling cascade by unlabeled insulin and FITC-insulin. WT MEFs were serum-starved for 2 h and stimulated with 100 nM unlabelled or FITC-labelled insulin for the indicated times. Cells were harvested and phosphorylation of signalling components determined by immunoblotting. C. WT and PKCε^−/−^ MEFs grown in glass-bottomed 96 well plates were serum-starved for 2 h and stimulated with 100 nM FITC-insulin for 10 min at 37°C. Cells were acid-washed, fixed, stained with DAPI, and insulin uptake analyzed by automated microscopy, on a per cell basis, based on internal cellular fluorescence intensity. Data are means ± range from 2 independent experiments. D. WT and PKCε^−/−^ MEFs were serum starved for 2 h and stimulated with 100 nM FITC-Insulin for 10 min at 37°C. Cells were acid-washed, trypsinized, fixed and insulin uptake analyzed by FACS. A representative FACS plot is shown. Means ± SEM of FACS analyses from at least 4 independent experiments carried out using two pairs of MEF cell lines. t-test: *p<0.05.

Our previous work showed that PKCε^−/−^ mice displayed decreased insulin clearance *in vivo* and that primary hepatocytes isolated from PKCε^−/−^ mice exhibited reduced insulin uptake [4]. To investigate the mechanisms involved we generated three independent pairs of mouse embryonic fibroblast (MEF) lines from WT and PKCε^−/−^ animals. The presence or absence of PKCε expression was confirmed by PCR (not shown) and immunoblot analysis (Fig. 1A). To determine whether PKCε^−/−^ MEFs also exhibit a defect in insulin uptake, FITC-labelled insulin was used to analyse uptake by automated fluorescence microscopy. Firstly, the ability of FITC-labelled insulin to stimulate insulin signal transduction in an equivalent manner to unlabeled insulin was determined (Fig. 1B), confirming that the labelled molecule was able to bind the insulin receptor. Quantification of internalized insulin revealed a significant reduction in insulin uptake in PKCε^−/−^ MEFs (Fig. 1C). A second approach using FACS analysis to quantify FITC-insulin uptake gave similar results (Fig. 1D).

### Insulin Receptor Phosphorylation is Reduced in PKCε^−/−^ MEFs

**Figure 2:**
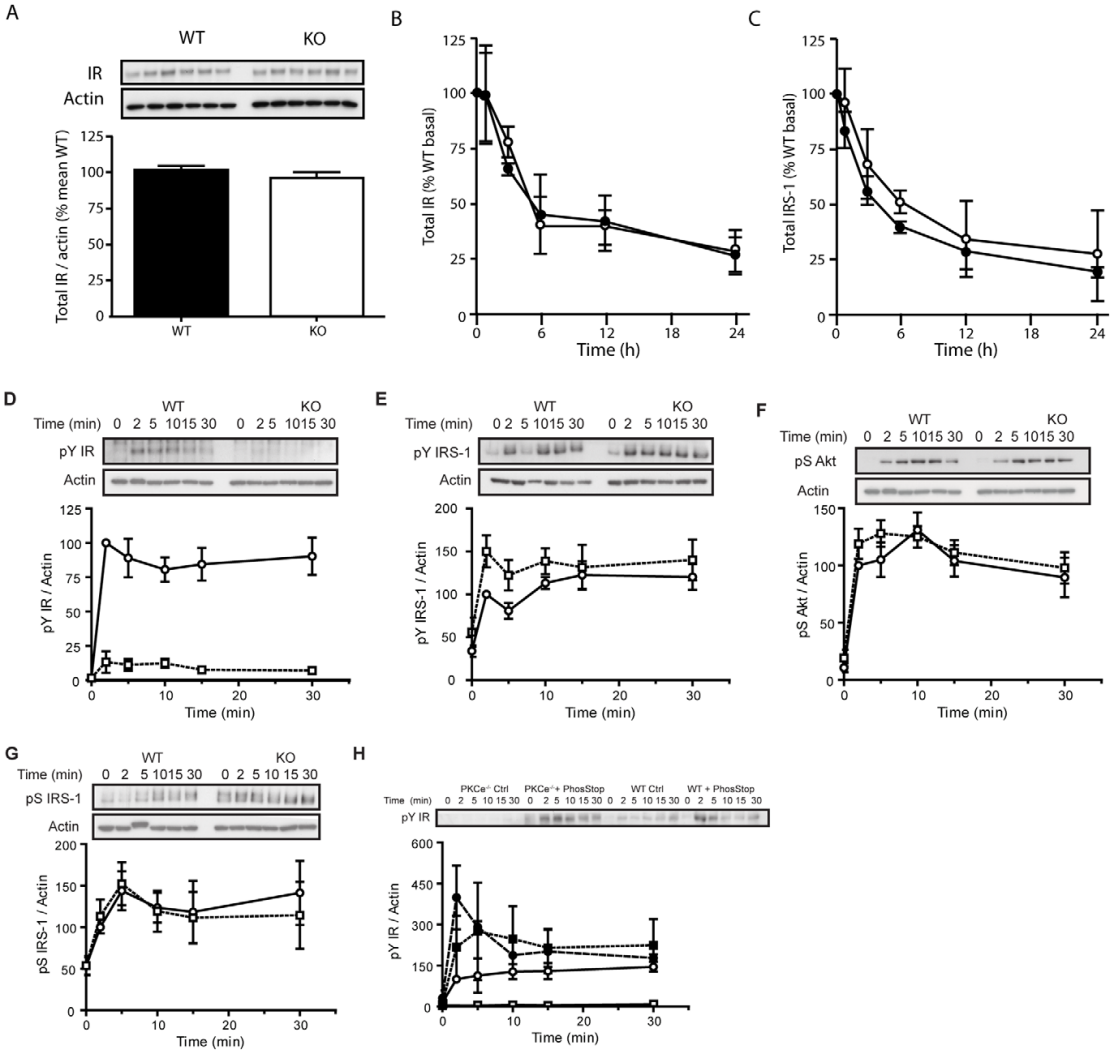
Proximal insulin signalling in WT and PKCε^−/−^ MEFs. A. Insulin receptor expression was assessed by immunoblotting, after cells under basal conditions were extracted and equal amounts of protein were subjected to SDS-PAGE. Data are means from 3 independent experiments. B,C. Insulin receptor and IRS-1 protein stability in the presence of chronic insulin were determined in WT (closed circles) and PKCε^−/−^ (open circles) MEFs treated with 1 µM insulin in the presence of 50 µg/mL cycloheximide for the indicated times. Data are means from 5 independent experiments carried out in duplicate. For the investigation of acute signalling, WT (closed circles) and PKCε^−/−^ (open squares) cells were serum-starved for 2 h and stimulated with 100 nM insulin for the indicated times, prior to cell lysis and immunoblotting. D. Phospho-Y1162/1163 and total insulin receptor; t-test: area under the curve WT vs PKCε^−/−^ MEFs P<0.001). E. Phospho-Y612 and total IRS-1. F. Phospho-S473 and total Akt. G. Phospho-S636/639 IRS-1. Data are means ± SEM from 3 or more independent experiments in 3 sets of MEF cell lines. H. MEFs were preincubated without (open symbols) or with (closed symbols) phosphatase inhibitors for 20 min prior to insulin stimulation, and insulin receptor Y1162/1163 phosphorylation determined by immunoblotting. Results shown are means from 2 independent experiments.

WT and PKCε^−/−^ cells exhibited similar levels of insulin receptor protein (Fig. 2A), and despite the reduced rate of receptor internalization, receptor halflife was not different in PKCε^−/−^ cells compared to WT cells upon chronic insulin stimulation (Fig. 2B). IRS-1 levels did tend to remain higher in the absence of PKCε (Fig. 2C, ANOVA: P = 0.085 over 24 h), suggesting some effects of kinase deletion may be exerted on downstream signalling. MEF cell lines were stimulated with 100 nM insulin over a 30 min time course to determine the effects of PKCε deletion on insulin signal transduction. PKCε^−/−^ MEFs displayed significantly reduced receptor phosphorylation at Y1162/63, compared to WT cells (Fig. 2D). In contrast however, downstream insulin signalling was not defective. Phosphorylation of IRS-1 at Y612 in fact tended to be increased above WT (Fig. 2E), while Akt S473 phosphorylation was not found to be different (Fig. 2F). Although individual pairs of WT and KO MEFs exhibited differing levels of inhibitory IRS-1 S636/639 phosphorylation, overall this did not correlate with the presence or absence of PKCε (Fig. 2G). Incubation of MEFs in the presence of phosphatase inhibitors increased subsequent insulin-stimulated insulin receptor Y1162/3 phosphorylation to similar levels in WT and PKCε^−/−^ cells, suggesting that there was no intrinsic defect in receptor tyrosine kinase activity in PKCε^−/−^ MEFs (Fig. 2H).

### PKCε^−/−^ MEFs Exhibit Altered Insulin Receptor Localization

**Figure 3:**
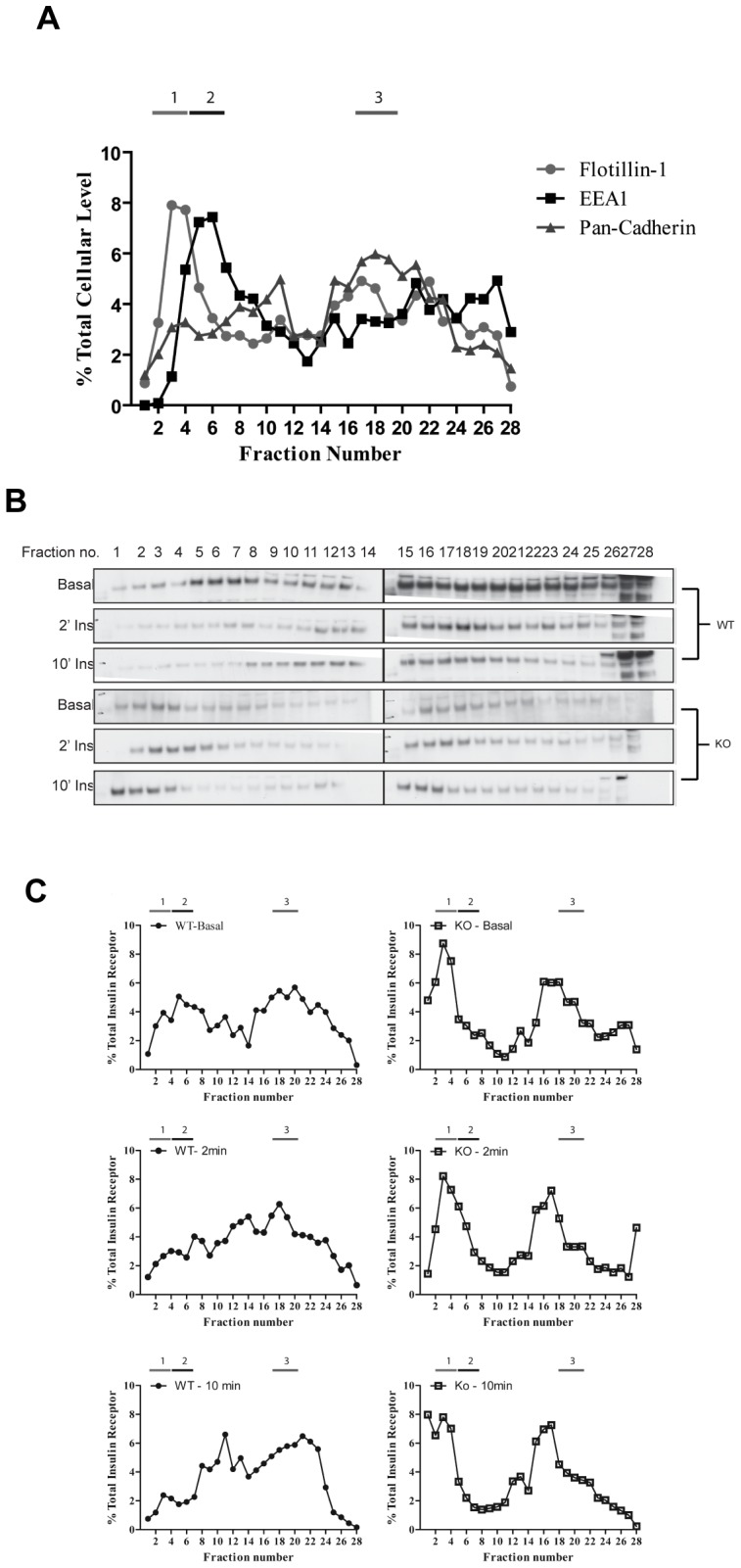
Insulin receptor subcellular fractionation in WT and PKCε^−/−^ MEFs. A. Cell extracts were separated by centrifugation using a continuous Opti-Prep density gradient. The presence of the markers flotillin1, EEA1 and pan-cadherin were determined in each fraction by immunoblotting and the means from two independent experiments shown. B. WT and PKCε^−/−^ MEFs were serum starved for 2 h and stimulated with 100 nM insulin for 2 or 10 min. Cells were extracted by nitrogen cavitation and fractionated as in A. Insulin receptor localisation was determined by immunoblotting (B) and mean levels in each fraction calculated from densitometry of 2 independent experiments (C).

We next explored whether the basal and insulin-stimulated cellular localization of the insulin receptor was altered by PKCε deletion. Subcellular fractionation of MEFs was performed by iodixanol density gradient centrifugation and the presence of subcellular marker proteins used to characterize the fractions. Flotillin1, a marker of membrane microdomains, was enriched in fractions 3–4 (Fig. 3A, peak 1). Early endosomal antigen 1 (EEA1), a marker for early endosomes, was most highly enriched within fractions 5–6 (Fig. 3A, peak 2). Pan-cadherin was employed as a general plasma membrane marker and exhibited a relatively broad distribution, with major peaks at fraction 11 as well as fractions 17–19 (Fig. 3A, peak 3). The distribution of these markers was similar in both WT and PKCε^−/−^ MEFs. There was however, a striking difference in localization of the insulin receptor in the basal state (Fig. 3B,C), with the receptor of PKCε^−/−^ MEFs found to be highly enriched in peak 1, coinciding with the flotillin1-enriched fractions of the gradient, compared to a broader distribution observed in WT MEFs. The insulin-induced redistribution of the receptor was also perturbed in PKCε^−/−^ MEFs. Upon insulin treatment, the receptor became relatively enriched in fractions 11–14 from WT cells especially after 10 min, whereas this was not observed in fractions from PKCε^−/−^ cells (Fig. 3B,C). This is consistent with reduced internalization of the insulin receptor in the absence of PKCε.

### Reconstitution of PKCε in MEFs Alters Insulin Receptor Localization

**Figure 4:**
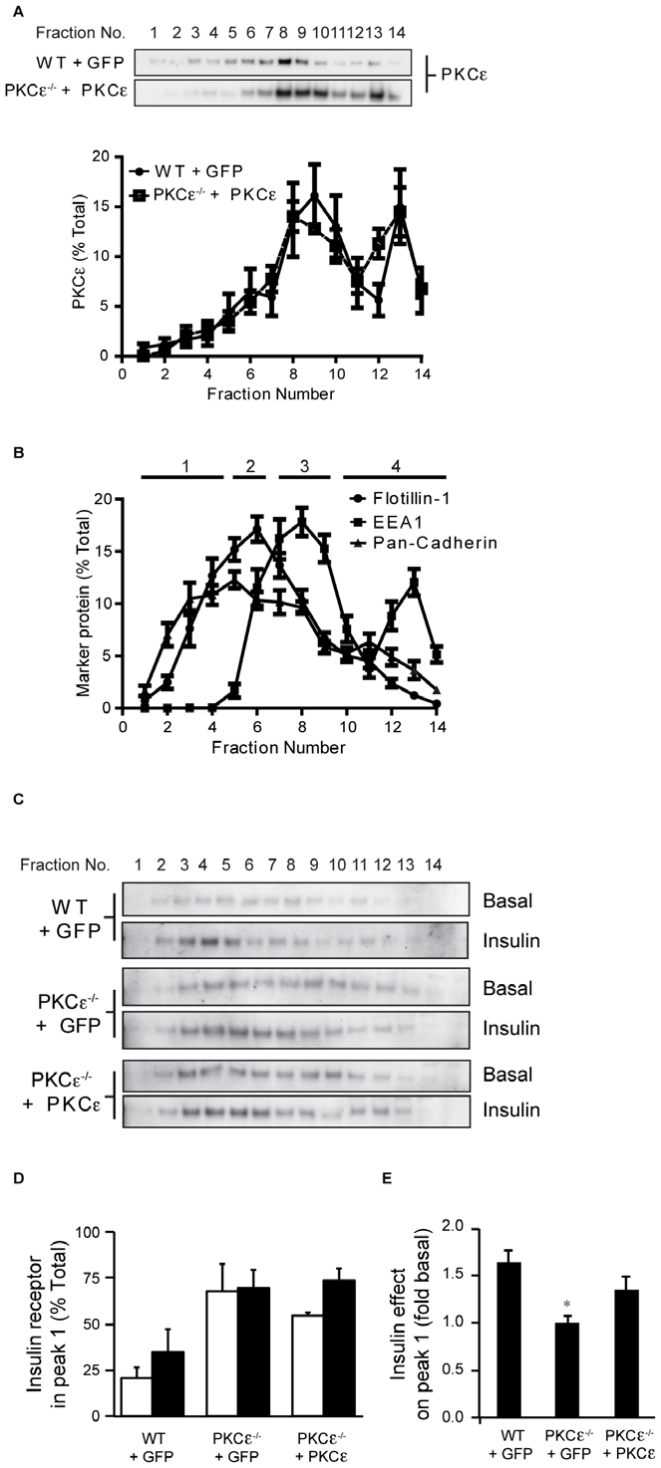
PKCε and insulin receptor localization in MEFs after reconstitution of PKCε. Adenovirus-infected WT MEFs overexpressing GFP and PKCε^−/−^ MEFs re-expressing PKCε were serum-starved for 2 h and stimulated with 100 nM insulin for 10 min at 37°C as indicated. Cells were disrupted by nitrogen cavitation and extracts subjected to Opti-Prep density gradient centrifugation. A. PKCε distribution was analyzed by immunoblotting gradient fractions from two independent experiments. B. The elution of flotillin1, EEA1 and pan-cadherin are shown as means from 2 independent experiments. C. The distribution of the insulin receptor from WT and PKCε^−/−^ MEFs overexpressing GFP, or PKCε^−/−^ MEFs reconstituted with PKCε, under basal and insulin-stimulated conditions. D. The relative amount of insulin receptor present in the first peak observed in gradient fractions. Data are means from 3 independent experiments performed in different pairs of MEF cell lines. E. The effect of insulin on WT and PKCε^−/−^ MEFs overexpressing GFP, or PKCε^−/−^ MEFs reconstituted with PKCε. t-test: *P<0.025 PKCε^−/−^ versus WT MEFs overexpressing GFP.

We then examined the effects of PKCε reconstitution on insulin receptor localization in MEFs, using acute adenovirally-mediated PKCε expression. PKCε^−/−^ MEFs were infected with recombinant adenovirus for the overexpression of PKCε, while virus mediating GFP overexpression was used as a control in WT and PKCε^−/−^ cells. Subcellular fractionation indicated that the distribution of overexpressed PKCε was similar to that of the endogenous kinase observed in WT MEFs (Fig. 4A). Although less gradient fractions of larger volume were collected in these experiments, the relative distribution of the cell compartment markers flotillin1, EEA1 and pan-Cadherin (Fig. 4B) was similar to that previously observed in cells not infected with adenovirus (Fig. 3). In PKCε^−/−^ MEFs expressing GFP under basal conditions, a greater proportion of the insulin receptor was again observed in earlier fractions (corresponding to peak 1 in Fig. 4B), compared to WT MEFs expressing GFP (Fig. 4C). This was more clearly demonstrated when the proportion of the total insulin receptor located in peak 1 was determined by densitometry (Fig. 4D), which showed that PKCε-deficient cells exhibited up to 3-fold more receptor in these fractions. This analysis also confirmed that insulin had no effect on receptor localization in PKCε^−/−^ cells expressing only GFP, while in WT MEFs insulin treatment led to the recruitment of the receptor to peak 1 (Fig. 4D). Although basal receptor localization in MEFs reconstituted with PKCε still more closely resembled that in control PKCε^−/−^ cells rather than WT cells (Fig. 4D), sensitivity to insulin was partly restored (Fig. 4D,E), with a greater than 30% increase in receptor levels observed in these fractions. This effect was no longer significantly different to that in WT cells (Fig. 4E).

**Figure 5:**
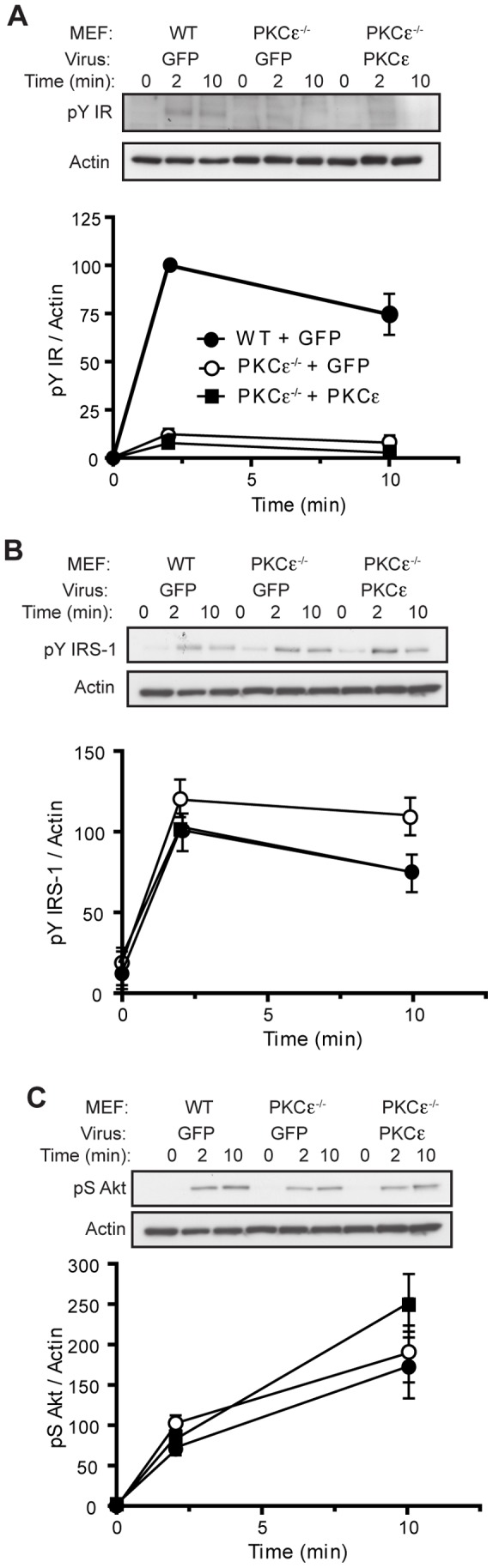
Insulin signalling in MEFs after reconstitution of PKCε. MEFs were infected with recombinant adenoviruses and stimulated with insulin as in Fig. 4. Lysates were analysed by SDS-PAGE and immunoblotting for phospho-Y1162/1163 (A), phospho-Y612 (B) and phospho-S473 (C) as for Fig. 2.

In contrast to these effects on insulin receptor redistribution, restoration of PKCε in MEFs did not restore insulin receptor phosphorylation to levels observed in WT cells (Fig. 5A) and did not affect downstream signalling at the level of IRS-1 or Akt (Fig. 5B,C).

### Ablation of PKCε does not Alter Membrane Morphology or Fluidity

**Figure 6:**
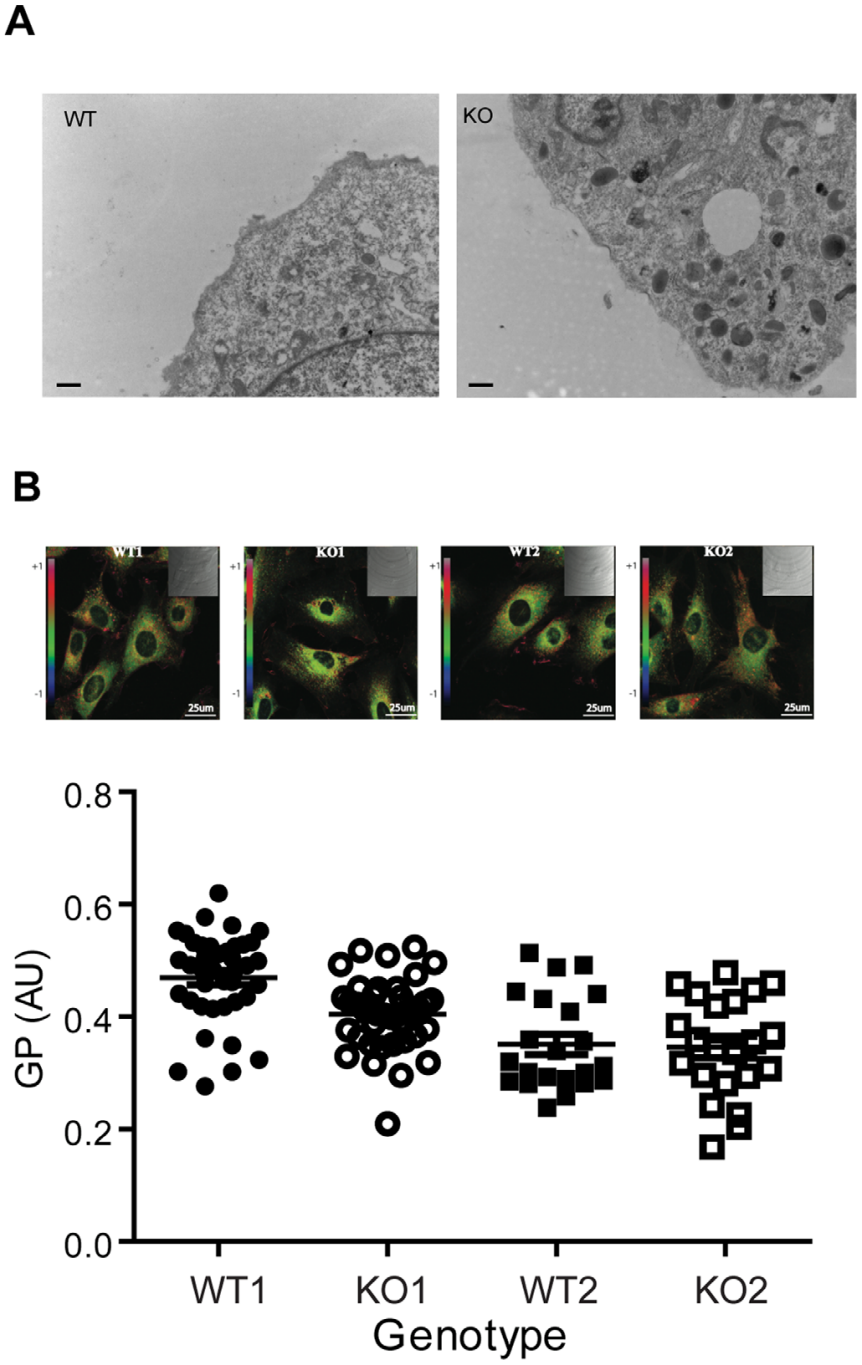
Membrane morphology and lipid order in WT and PKCε^−/−^ MEFs liver. A. TEM images of WT and PKCε^−/−^ MEFs, representative of 13 and 14 cells analysed respectively. B. Quantitative imaging of membrane lipid order in WT and PKCε^−/−^ MEFs using the polarity-sensitive membrane dye Laurdan. Representative generalized polarization (GP) images are shown of 20–40 cells analysed in each group (upper panel). The mean GP was calculated for the outer membrane area region of each cell (lower panel).

We next investigated whether the differences in insulin receptor localization and redistribution were associated with differences in membrane morphology by subjecting MEFs to TEM. No difference in gross morphology of the plasma membrane was observed between the WT and PKCε^−/−^ MEFs (Fig. 6A). We also determined the lipid order of the plasma membrane by two-photon microscopy of cells which had been incubated with the polarity-sensitive membrane dye Laurdan. Direct examination of the organization of the membrane in this way did not indicate any PKCε-dependent changes in fluidity (Fig. 6B).

### CEACAM1 Expression in MEFs is Dependent on PKCε

**Figure 7:**
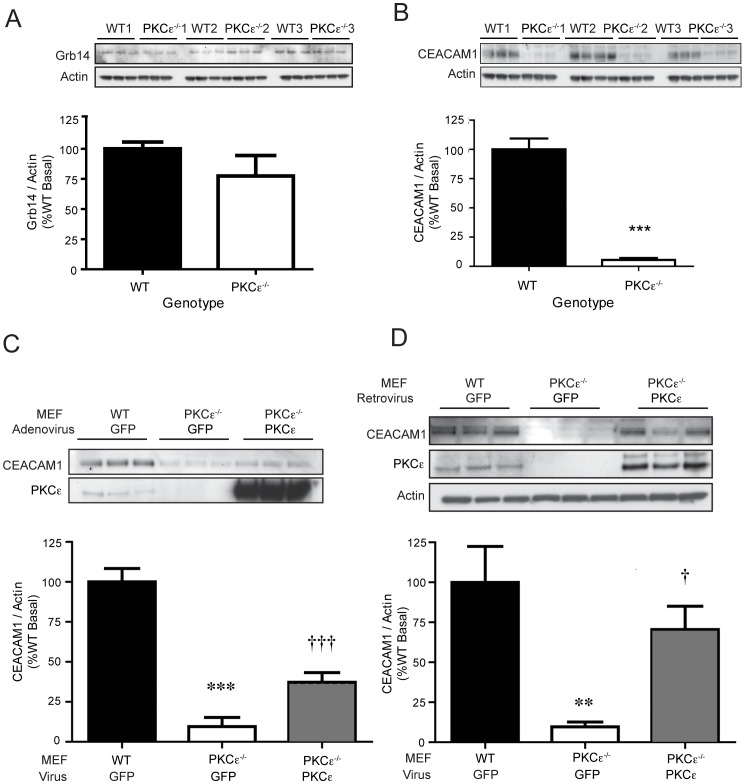
Grb14 and CEACAM1 expression in WT and PKCε^−/−^ MEFs. Grb14 (A) and CEACAM1 (B) protein levels in lysates from WT and PKCε^−/−^ MEFs were determined by immunoblotting, and expressed as a percentage of the corresponding WT MEF cell line. Data are means ± SEM; (***p<0.001 vs WT (n = 3–6 independent experiments in each MEF pool). C. WT MEFs were infected with GFP-expressing control adenovirus and PKCε^−/−^ MEFs infected with either GFP- or PKCε-expressing adenovirus for 48 h. CEACAM1 expression determined by immunoblotting and expressed as a percentage of WT. Data are means ± SEM; t-test: ***p<0.001 vs WT GFP; ^†††^ = p<0.001 vs PKCε^−/−^ GFP (n = 3–6 independent experiments performed in two separate MEF pools). D. WT MEFs were infected with GFP control retrovirus and PKCε^−/−^ MEFs with GFP control or PKCε-expressing retrovirus for 5–10 passages. MEFs were harvested and CEACAM1 expression analyzed by immunoblotting. Data are means ± SEM from 3 independent experiments performed in two separate MEF pools; t-test: **p<0.01 vs WT GFP; ^†^ = p<0.05 vs PKCε^−/−^ GFP.

To determine whether the alterations in insulin receptor signalling and localization observed in PKCε-deficient MEFs were due to changes in the expression of known insulin receptor regulatory proteins, we examined the levels of Grb14 and CEACAM1 in these cells. These binding partners have been reported previously to affect insulin receptor phosphorylation or internalization [6], [12]. Grb14 protein expression levels were not consistently different between WT and PKCε^−/−^ MEF cell lines (Fig. 7A). However, all PKCε^−/−^ cell lines exhibited a major reduction in CEACAM1 expression compared to WT cells (Fig. 7B). When PKCε was reconstituted by infection of the cells with recombinant adenovirus for 48 h, CEACAM1 expression was restored in PKCε^−/−^ MEFs to levels over 30% of those in WT MEFs (Fig. 7C). We also examined whether longer term restoration of PKCε to more physiological levels in MEFs would further enhance CEACAM1 expression, by generating stable cells lines using a retroviral vector. When PKCε protein expression was induced in PKCε^−/−^ MEFs to 1.7-fold that observed in WT MEFs, CEACAM1 protein levels increased to approximately 70% of those in WT cells (Fig. 7D). In stable cell lines passaged for longer, CEACAM1 levels returned to levels not distinguishable from those in WT MEFs (data not shown).

## Discussion

We previously demonstrated that PKCε ablation in mice reduces hepatic insulin clearance [4]. Here we show that in the absence of PKCε, insulin uptake and insulin receptor autophosphorylation, localization and trafficking in MEFs is perturbed, and that this is not due to differences in cellular morphology or membrane fluidity. Instead, alterations in the distribution of the insulin receptor are associated with reduced expression of the insulin receptor substrate CEACAM1, previously shown to be required for insulin receptor internalization [6]. Acute reconstitution of PKCε partly restores both CEACAM1 expression and receptor trafficking, while autophosphorylation remains impaired.

The insulin receptor has previously been reported to interact with specific PKC isoforms in NIH-3T3 cells (PKCα, PKCδ and PKCζ), which affected the intracellular routing of the receptor to regulate sorting between degradative and retroendocytotic pathways [13]. Here, PKCε deletion resulted in a reduction of insulin uptake by MEFs, in agreement with our previous data from isolated primary hepatocytes [4]. This was, however, independent of any effects on insulin receptor protein levels or half-life. Consistent with a specific effect on insulin uptake, overall plasma membrane structure and fluidity also did not appear to be perturbed in PKCε^−/−^ MEFs.

The data obtained using FITC-labelled insulin is in good agreement with our previous work showing a reduced rate of I^125^-labelled insulin uptake by primary PKCε^−/−^ hepatocytes [4], and we have now extended those findings by investigating insulin receptor trafficking. Upon density gradient centrifugation and fractionation, a larger proportion of the insulin receptor from PKCε^−/−^ MEFs was observed in fractions enriched for flotillin-1, suggesting localization within membrane microdomains. In addition, insulin stimulation had little effect on receptor distribution, in contrast to the effects of the hormone on the receptor in fractions from WT MEFs. This was associated with markedly reduced levels of receptor autophosphorylation. Because the phosphorylation of downstream signalling components was not defective, and phosphatase treatment of MEFs was able to restore receptor phosphorylation, these findings may indicate rapid dephosphorylation specifically of the insulin receptor after initial activation of its intrinsic tyrosine kinase activity and phosphorylation of IRS-1. Signalling may also be enhanced by the prior location of the receptor to flotillin-enriched lipid rafts [14] despite the reduced autophosphorylation levels. While subtle, the moderately increased stability of IRS-1 may also be related to the tendency for IRS-1 tyrosine phosphorylation to be increased in PKCε^−/−^ cells, although this did not appear to be transmitted to Akt activation. A discrepancy between insulin receptor and IRS-1 tyrosine phosphorylation has also been observed in the absence of Grb14, under which conditions dephosphorylation of the receptor is promoted while downstream signalling is in fact enhanced [12]. However, PKCε ablation did not reduce Grb14 expression in the MEF cell lines, suggesting other factors are involved.

The difference in insulin receptor redistribution led us to examine CEACAM1 protein levels in MEFs. CEACAM1 plays a role in insulin receptor endocytosis [15], and liver-specific overexpression of a defective CEACAM1 mutant has been shown to result in markedly reduced hepatic insulin clearance in mice [6]. Furthermore, activation of PKC isoforms with phorbol ester can enhance CEACAM1 expression [16]. We now showed that PKCε^−/−^ MEFs expressed CEACAM1 at far lower levels than WT cells, and that two different approaches resulting in reconstitution of PKCε restored CEACAM1 protein, implicating PKCε as a regulator of CEACAM1 expression. The fact that longer term retrovirally-mediated PKCε reconstitution, even at lower levels than observed with acute adenovirus treatment, was able to restore CEACAM1 more fully may indicate that this effect is indirect, and may be an adaptation to the presence of PKCε which confers a survival or proliferative advantage on the MEFs.

Taken together with the altered localization of insulin receptor in unstimulated PKCε^−/−^ MEFs, and the failure of insulin to promote insulin receptor redistribution, these findings suggest that the effect of PKCε deletion on insulin receptor trafficking could be mediated by a loss of CEACAM1. We speculate that this reduces the ability of the receptor to be recruited from lipid microdomains into clathrin coated pits for endocytosis, in agreement with the proposed role of CEACAM1 [7]. The effects of PKCε re-expression on CEACAM1 restoration and insulin receptor trafficking in MEFs further supported a role for PKCε and CEACAM1 in receptor internalization. Although PKCε reconstitution did not relocalize the insulin receptor to precisely the same subcellular fractions in which it was observed in uninfected WT MEFS, evidence for a partial restoration of insulin sensitivity in terms of relocalization was observed. Re-expression of PKCε did not, however, lead to restoration of insulin receptor autophosphorylation, suggesting either that distinct mechanisms underlie the changes in insulin receptor trafficking and phosphorylation level.

Inhibition of insulin receptor internalization has previously been shown to selectively attenuate insulin signalling pathways. Consistent with the data presented here, a reduction in clathrin-mediated endocytosis of the receptor, mediated by overexpression of a dominant-interfering dynamin mutant, did not significantly affect IRS-1 tyrosine phosphorylation or Akt activation [17]. However, insulin receptor autophosphorylation was also preserved, again suggesting that the reduced phosphorylation we have observed is due to other effects of PKCε deletion, such as localization in a specific membrane compartment promoting tyrosine dephosphorylation.

It is important to note that the experiments reported here concerned different clones of MEFs derived from PKCε^−/−^ mice, rather than primary hepatocytes, which would be more closely relevant to the study of insulin internalization. The mechanisms involved in insulin receptor trafficking in these different cell types could be different. However, insulin uptake is defective in both cell types, indicating that the defect in insulin receptor trafficking is associated with the deletion of PKC independent of the cell type. Interestingly, mice with a null mutation of CEACAM1 exhibit increased triglyceride storage in the liver, which is similar to short term effects observed in PKCε^−/−^ mice fed a high fat diet [9]. Although the effects of PKCε deletion on insulin clearance and hepatic lipid storage are milder than those of CEACAM1 mutation or deletion, and do not result in secondary insulin resistance and steatohepatitis [9], these observations are consistent with the involvement of PKCε and CEACAM1 in a common mechanism which regulates insulin receptor trafficking and function.

In summary, the data presented here support the hypothesis that ablation of PKCε alters insulin receptor trafficking, and that this could be mediated through alterations in the expression of the insulin receptor substrate, CEACAM1.

## Supporting Information

Methods S1 Methodological details of antibodies used, fluorescence microscopy, fluorescence-activated cell sorting, transmission electron microscopy, and 2-photon microscopy are given.
(DOCX)
