# Supplementary material for: Protein Kinase Cε Modulates Insulin Receptor Localization and Trafficking in Mouse Embryonic Fibroblasts
Source: PLoS One. 2013 Mar 1;8(3):e58046. doi: 10.1371/journal.pone.0058046 (PMC3585804; doi:10.1371/journal.pone.0058046)
Supplement: Methods S1 — Methodological details of antibodies used, fluorescence microscopy, fluorescence-activated cell sorting, transmission electron microscopy, and 2-photon microscopy are given. (DOCX) [file pone.0058046.s001.docx]

**Supporting Information**

**Methods S1**

*Antibodies*–PKCε, caveolin 1, flotillin-1, Rab 11 and insulin receptor (β-subunit) antibodies were obtained from BD Biosciences (Sydney, NSW, Australia). Insulin receptor (β-subunit), Akt (phospho Ser473 and total), IRS-1 (phospho Ser636/639), Early Endosome Antigen 1 (EEA1), SOCS3 and pan-cadherin antibodies were from Cell Signalling Technology, Inc. (Danvers, MA). IRS-1 (total) and Grb14 antibodies were also purchased from Millipore Merck Ltd. (Kilsyth, VIC, Australia). Insulin receptor (phospho Tyr1162/1163), IRS-1 (phospho Tyr612) and β-actin antibodies were from Sigma-Aldrich Pty. Ltd. (Sydney, NSW, Australia). CEACAM-1 antibody was a kind gift from Professor Andre Marette (Montreal Diabetes Research Centre, Canada).

*Determination of Insulin Uptake by Fluorescence Microscopy*–MEFs were grown to 90-95% confluency in poly-L-lysine coated (5 *μ*g/mL) 96-well glass bottom plates. After serum-starvation for 2 h, cells were incubated with 100 nM FITC-labelled insulin for 10 min at 37°C. Cell surface-bound insulin was removed by incubating the cells in an ice-cold acid wash solution (0.2 M acetic acid, 0.5 M NaCl, pH 4.5) with gentle shaking for two 5 min washes followed by two washes with ice-cold phosphate-buffered salin (PBS) for 5 min each. Cells were fixed with 4% (w/v) paraformaldehyde for 15 min at 37 °C and nuclei stained with 4'-6-Diamidino-2-phenylindole (DAPI). Quantitative analysis of FITC-insulin uptake was performed using an automated image acquisition and analysis system (Image Xpress Micro (IXM), Molecular Devices, Sunnyvale, CA). Nine images were collected from each well, averaging 20-30 cells per image. The average integrated intensity of the FITC-insulin signal per cell was calculated and data expressed as a percentage of uptake relative to WT cells.

*Determination of Insulin Uptake by fluorescence-activated cell sorting (FACS)–*MEFs were serum-starved for 2 hours prior to insulin stimulation. For the last 20 min, MEFs were incubated with 50 µM monensin to block receptor recycling. Insulin receptor internalization was stimulated with the addition of 100 nM FITC-insulin for 10 min at 37°C, after which cells were washed twice with ice-cold PBS for 5 min with gentle shaking. FITC-insulin was stripped from the cell surface with three 5 min acid washes (DMEM, 0.2% (w/v) BSA adjusted to pH 4.5 with HCl) with gentle shaking. This was followed by three 5 min ice-cold PBS washes before cells were trypsinised (0.25% (w/v) trypsin in 1 mM EDTA) on ice for 15 min. Cells were centrifuged at 1,200*g* for 5 min at 4°C, and washed twice in ice-cold FACS buffer (PBS, 0.1% (w/v) BSA, 0.1% (w/v) sodium azide) and fixed in FACS buffer containing 2% (w/v) paraformaldehyde, in the absence of light at 4°C for 16 h. Cells were centrifuged at 1,200*g* for 5 min at 4°C and washed three times in ice-cold FACS buffer, before being passed through a 70 µm filter and analyzed using a FACSCanto flow cytometer (BD Biosciences) and FlowJO software (Tree Star, Ashland, Oregon).

*Transmission electron microscopy (TEM*)–MEFs were fixed with 4% (w/v) paraformaldehyde, 0.1% (w/v) glutaraldehyde in 0.12M phosphate buffer, pH 7.4, and placed in 1% (v/v) osmium tetroxide solution for 15-30 min before washing six times for 5 min each in maleate buffer (pH 5). Cells were treated with 1% (v/v) uranyl acetate overnight at 4°C, dehydrated through a series of ethanol solutions (50%, 70%, 85%, 95% and 100%) and infiltrated with resin (polybed 812) which was polymerized at 60°C in a BEEM capsule. Ultrathin sections were cut for examination by TEM as described ([1](#_ENREF_1)).

*Analysis of membrane fluidity by 2-Photon microscopy of Laurdan stained cells*–MEFs were labelled with 10 µM Laurdan (Molecular Probes) and fixed with 4% (w/v) paraformaldehyde. Laurdan was excited at 800nm and emission intensities were simultaneously recorded in the range of 400-460nm and 470-530nm using a Leica DM IRE2 2-photon microscope. Intensity images were converted into Generalized Polarization (GP) images (WiT software). The outer membrane area of the cell was defined as the region of interest (ROI) and the mean GP of the ROI determined using ImageJ software (National Institutes of Health, Bethesda, MA).

**References**

1. Rubio, M. E., Gudsnuk, K. A., Smith, Y., and Ryugo, D. K. (2008) *Neuroscience* **154**, 99-113
